# A Numerical Study on the Crashworthiness of Corrugated Conical Tubes with Small Semi-Apical Angles and Their Influence Mechanism

**DOI:** 10.3390/biomimetics10010029

**Published:** 2025-01-06

**Authors:** Yiheng Song, Qinyu Lin, Jinxiang Chen, Tidong Zhao

**Affiliations:** School of Civil Engineering, Key Laboratory of Concrete and Prestressed Concrete Structures of the Ministry of Education, Southeast University, Nanjing 211189, China; utsongyih@gmail.com (Y.S.);

**Keywords:** biomimetic, energy absorption, crashworthiness, semi-apical angle, corrugation

## Abstract

To develop a new type of biomimetic single-cell and multi-cell energy-absorbing box (tube) featuring conical tubes at the intersection of cell walls, it is necessary to address the issue of large bottom-space requirements in current conical energy-absorbing tubes with superior crashworthiness due to their large semi-apical angles. This study proposes adding corrugations to conical tubes with small semi-apical angles and modifying the bottom by replacing the last one or two inclined corrugations with vertical ones. Finite element simulation results show that, compared to conventional conical tubes, adding corrugations reduces the optimal semi-apical angle of conical tubes by 5°, with the optimal range being 5–10°. Furthermore, the modification method of replacing inclined corrugations with vertical ones effectively mitigates the challenges of increasing peak crushing force and large end-peak crushing force as the semi-apical angle increases. This structural optimization lays a foundation for the development of new biomimetic single-cell and multi-cell energy-absorbing boxes (tubes) incorporating conical tubes.

## 1. Introduction

Buffer energy-absorbing structures have been widely utilized in transportation equipment [1], logistics packaging, construction and other fields [2]. Cars, which are commonly used in daily life, have energy-absorbing tubes in the front longitudinal rail to buffer frontal collisions [3,4].

**Figure 1:**
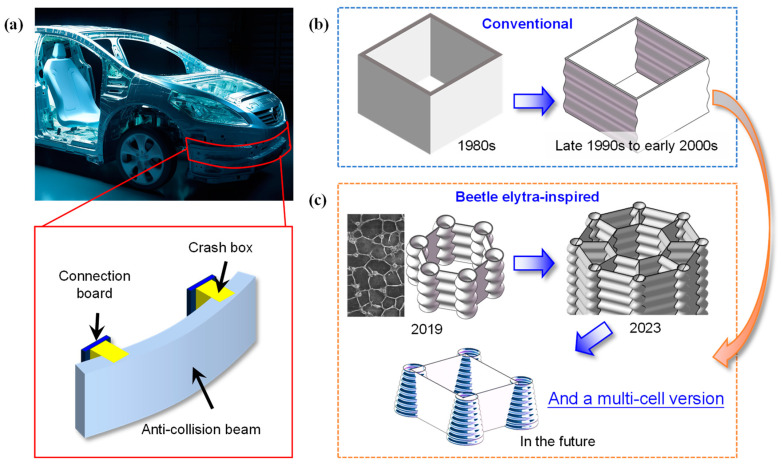
Development of types of automotive crash boxes and their energy-absorbing structures. (**a**) Application of single-tube crash beams; (**b**) conventional crash boxes; (**c**) bio-inspired crash box structures proposed by our group, featuring single-cell and multi-cell plate–tube combinations.

Crash boxes (Figure 1a) were first applied in the 1980s, marking the beginning of their design and research. Initially, they were predominantly simple single-cell tubes. While these designs were easy to manufacture, their energy absorption efficiency was low, resulting in limited effectiveness [5]. In the late 1990s to early 2000s, corrugated structures were introduced (Figure 1b), which spurred extensive discussion over a prolonged period. Subsequently, more innovative designs began to emerge. The first type involved introducing weak spots on thin-walled straight tubes, such as ridges [6,7], grooves [8] and holes [9], to guide controlled deformation in energy-absorbing tubes. The second type typically added longitudinal ribs or combined multiple tubes of varying sizes to create multi-cell [10], multi-tube [11] and hierarchical [12] configurations. The third type applied gradient adjustments to shape parameters [13] or supplemented the structure by filling tubes with foam [14]. In addition to structural design, efforts were made to optimize crashworthiness from material and spot-welding perspectives [15,16], and algorithms like metaheuristics and the slime mold algorithm were employed in design optimization [17,18]. Our group started by observing the microstructure of beetle elytra and discovered that, through evolution, a honeycomb-core layer with small trabeculae had formed beneath their exoskeleton (Figure 1c, scanning electron microscope image). Utilizing biomimetic approaches and further refinement, a novel bio-inspired structure—a beetle core unit crash box—was proposed in 2019 [19,20] (Figure 1c). Compared with conventional single-tube square crash boxes, this new design increased both energy absorption and efficiency by over three times while maintaining a similar initial peak crushing force (*IPCF*) [18]. Later, in 2023, a multi-cell bio-inspired energy-absorbing structure (Figure 1c) was developed as an alternative to the commonly used single-cell structures, aiming to reduce peak impact forces [21].

Our group will continue to enhance energy absorption by introducing further optimization to the single-cell and multi-cell bio-inspired models, specifically by replacing the uniform cross-section straight tube with a variable cross-section conical energy-absorbing tube, which holds great potential. Similar approaches have already been explored by many researchers. For example, Yang et al. [22,23] reported the crashworthiness of conical tubes with sandwich structures. Zhang et al. [24] proposed conical tubes with linear and nonlinear wall thicknesses. Attar and Kazemi [25] compared the peak crushing force and energy-absorbing capacity of conical tubes with different layer arrangements. It has been proven that, regardless of the section shape [26], structural complexity [10,27], or loading direction [28], conical structures have a lower *IPCF*, a smaller fluctuation amplitude of the force–displacement curve, a more stable crushing deformation mode, and a strong energy-absorbing capacity [26,29]. To date, the influence of structural parameters, such as length, wall thickness and semi-apical angle, on the crashworthiness of conical tubes has been clarified [24,30,31]. For example, increasing the semi-apical angle can significantly reduce the *IPCF*; a semi-apical angle that enables a low *IPCF* and high specific energy absorption (*SEA*) usually ranges from 10 to 15°. Therefore, the next step is to replace circular tubes with conical tubes in the aforementioned single-cell and multi-cell plate–tube energy-absorbing structures (Figure 1c), and develop a new type of bio-inspired plate–cone energy-absorbing box (tube).

**Figure 2:**
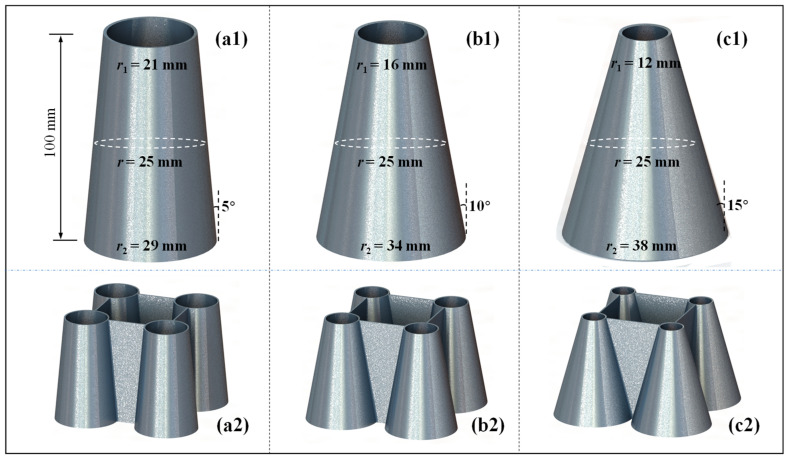
Conical tubes with different semi-apical angles and plate–cone crash boxes. (**a1**,**a2**) 5°; (**b1**,**b2**) 10°; (**c1**,**c2**) 15°.

Nevertheless, when the semi-apical angle is 10–15°, as the tube length increases, the difference between the diameter of the top and bottom sections of the conical tube can be substantial (Figure 2). This approach has disadvantages, such as requiring a large space for the tube bottom, which may limit its application and promotion. The second row of Figure 2 takes a simple square crush structure as an example and shows the combination effect of plates and conical tubes with different semi-apical angles. When the semi-apical angle reaches 10° and 15°, the bottom of the structure has already taken up a large space, which is difficult to apply to longer or more complex energy-absorbing tubes, such as in Figure 1c. In this regard, corrugations can be introduced to reduce the semi-apical angle of conical tubes. Mortazavi Moghaddam et al. [26] proved that, compared to the conventional square straight tube commonly used in automobiles, the corrugated conical tube (CCT) had a relatively moderate *IPCF* and a higher *SEA*, and its structure was more stable and controllable than a conical tube without corrugations. Similarly, Ahmadi et al. [32] demonstrated that CCTs could avoid global buckling. Additionally, Ahmadi et al. [30] and Alkhatib et al. [33] reported the impact of wall thickness, corrugation wavelength and corrugation amplitude on crashworthiness. Since corrugations also weaken the energy-absorbing capacity of a conical tube, its optimal semi-apical angle range should be different from that of a conventional conical tube. However, relatively little research has been conducted to date. Therefore, further research is needed on the influence of the semi-apical angle on the crashworthiness of CCTs.

This study proposes a novel design approach for corrugated conical tubes by introducing corrugations to conical tubes with small semi-apical angles and replacing the last one or two inclined corrugations at the bottom with vertical ones to optimize structural performance. This innovative design effectively addresses the issue of large bottom-space requirements in current conical energy-absorbing tubes with superior crashworthiness due to their large semi-apical angles. The findings provide a new perspective for developing bio-inspired single-cell and multi-cell energy-absorbing boxes (tubes) featuring conical tubes at the intersection of cell walls, laying a solid foundation for the design of compact and efficient energy-absorbing systems.

## 2. Methods

### 2.1. Design of CCTs

**Figure 3:**
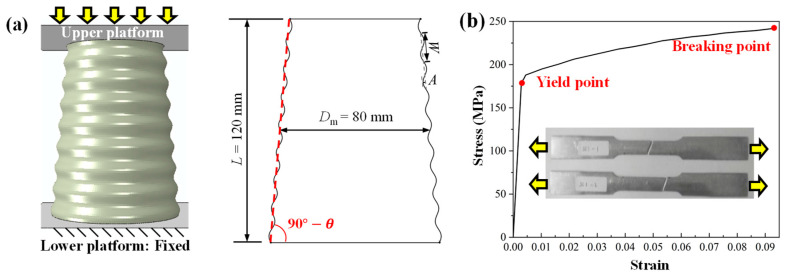
CCT and aluminum alloy. (**a**) Schematic of the structural parameters of CCTs, and (**b**) true strain–stress curve of aluminum alloy.

CCT models with a *θ* value of 0° (straight tube, as the control group), 5°, 7.5°, 10°, and 12.5° were considered in this paper and were coded as SA0, SA5, SA7.5, SA10 and SA12.5, respectively. The geometric parameters are shown in Figure 3a. The tube length *L* was fixed at 120 mm, and the diameter at the middle height *D*_m_ was fixed at 80 mm. The remaining structural parameters were determined as follows: the wall thickness *t* was 1.5 mm, corrugation wavelength *W* was 15 mm, and corrugation amplitude *A* was 1.25 mm (Figure 3a). The corrugation number of each tube was 8. CCTs use an aluminum alloy with excellent ductility (aluminum alloy 6063T5) as their material.

### 2.2. Indices for Evaluating Crashworthiness

Apart from the initial peak crushing force (*IPCF*) and maximum peak crushing force (*MPCF*), the following three indices are used to evaluate the crashworthiness of CCTs.

(1) Energy absorption 

*SEA* is the ratio of *EA* to the mass. Because the mass of each model in this paper is close to 130 g and the difference exists only in the value after the decimal point, the relationships and patterns shown by *SEA* are actually consistent with *EA*. Hence, only *EA* is chosen to be elaborated.

Energy absorption (*EA*) is used to characterize the ability of an energy-absorbing tube to dissipate crushing energy through plastic deformation:

(1) $$EA=\int_{0}^{l_{\max}}F\left(x\right)dx$$

where *F*(*x*) is the axial crushing force as a function of displacement *x* during the crushing process and *l*_max_ is the effective deformation length. In this paper, the lowest point before densification is taken as the failure point.

(2) Mean crushing force

The mean crushing force (*F*_m_) is the average compression force of the energy-absorbing tube within the effective deformation length:

(2) $$F_{m}=\frac{EA}{l_{\max}}$$

(3) Crushing force efficiency

The crushing force efficiency (*CFE*) is the ratio of *F*_m_ to *IPCF*, which is mainly used to evaluate the fluctuation amplitude of the force–displacement curve during the crushing of the energy-absorbing tube [34,35]:

(3) $$CFE=\frac{F_{m}}{IPCF}$$

### 2.3. Finite Element Method (FEM) and Validation

**Figure 4:**
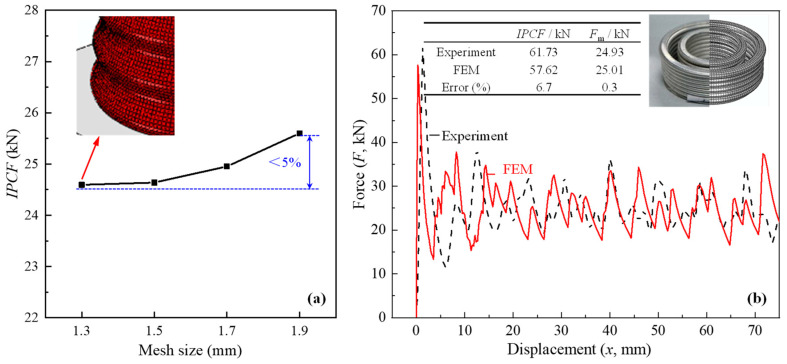
Mesh sensitivity analysis and FEM model validation. (**a**) Mesh sensitivity analysis; (**b**) comparison between FEM model and experiment.

The ABAQUS 2020 explicit solver was used to simulate the crushing process of CCTs. The CCT was placed between two rigid plates simulated with the R3D4 element. The bottom plate was completely fixed, while the top plate acted as a striker that applied the load and only had a vertical translational degree of freedom. Smooth displacement control was adopted for the loading process. A general contact algorithm with hard contact in normal behavior and a 0.2 friction coefficient in tangential behavior [30] was defined. The displacements and crushing force were extracted from the reference point of the top and bottom plates, respectively. Aluminum alloy 6063T5 was adopted, and its mechanical properties (Table 1) and stress–strain curve (Figure 3) were obtained experimentally by Fu et al. [36] (according to the ASTM E8M standards [37]). Isotropic elastic–plastic behavior was used to describe the stress–strain curve. Furthermore, since the application scenario is set to automotive collisions, the strain rate of the materials involved typically falls within the range of 10^1^–10^2^ s⁻^1^. Aluminum alloy, being strain-rate-insensitive within this range [28], does not require the consideration of rate effects in the simulations. The yield behavior of the material is defined using the von Mises criterion. The CCTs were divided by approximately 18,000 four-node, reduced-integration (S4R) shell elements. Each element has one integration point. Mesh sensitivity analysis was conducted (Figure 4a), which showed that when the mesh size was reduced to 1.3 mm, the calculation result was relatively stable and could be used for subsequent parameter studies.

Due to the similarity in FEM modeling steps and calculation methods between bitubal circular tubes and corrugated conical tubes (CCTs), this study selected the classical bitubal circular tubes as a benchmark model for finite element calibration. The finite element model of this paper was verified by simulating the compression experiment of Fu et al. [32] on a bitubal circular tube. The length of the bitubal circular tube was 100 mm, the diameters of the inner and outer tubes were 40 mm and 62 mm, respectively, and the wall thickness was 1 mm. Figure 4b compares the results of the experiment and numerical simulation. The force–displacement curves of the two were similar in shape, and both dropped sharply and fluctuated at a certain height after rising to the initial peak. The errors of *IPCF* and *F*_m_ between the experiment and simulation were less than 10%. From the perspective of deformation, the FEM model presented a ring deformation mode, which was also consistent with the experiment. Therefore, it can be concluded that the FEM model demonstrates high accuracy, with the modeling and simulation methods being reliable. Subsequently, it can be utilized to simulate the compression process of corrugated conical tubes (CCTs). Thus, the finite element model was deemed sufficiently accurate to reflect the influence of the semi-apical angle on the behavior of CCTs.

## 3. Results and Discussion

### 3.1. Deformation and Crashworthiness of CCTs with Different θ Values

In this section, the deformation modes, force–displacement curves, and crashworthiness characteristics of CCTs with different *θ* values were investigated, and their advantages and disadvantages were noted.

**Figure 5:**
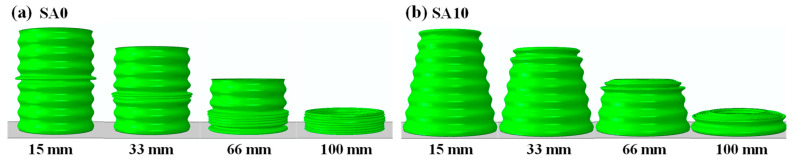
Deformation modes of corrugated straight and corrugated conical tubes under different compression levels. (**a**) Corrugated straight tube; (**b**) corrugated conical tube.

**Figure 6:**
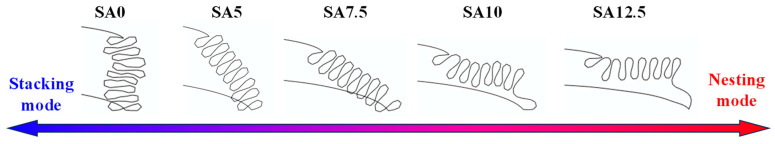
Deformation wireframe of each corrugated tube at compression displacement of 105 mm (half shown for symmetry).

**Figure 7:**
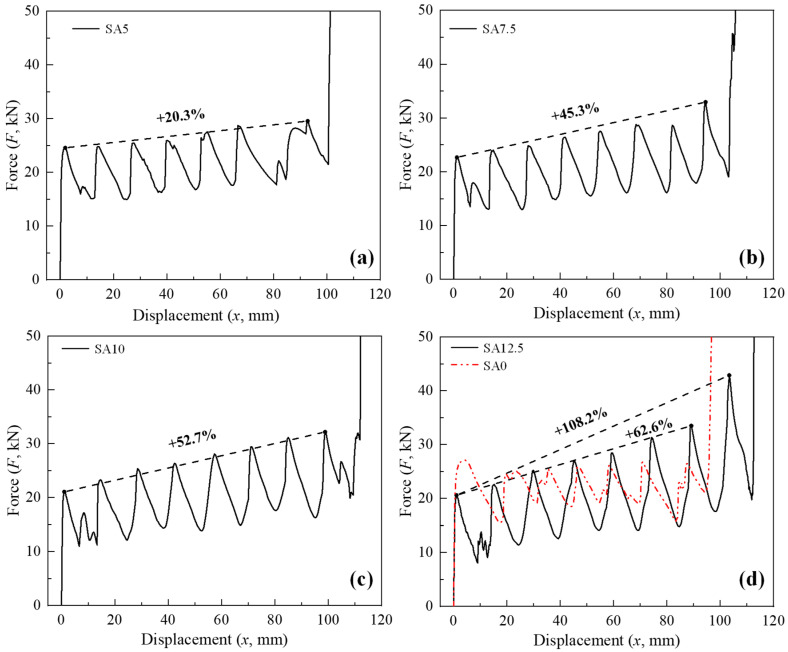
The force–displacement curves of CCTs with different *θ* values. (**a**) SA5; (**b**) SA7.5; (**c**) SA10; and (**d**) SA12.5 and SA0. Note that above the dotted line is the difference percentage of the initial and end peak crushing forces, indicating an increase in the peak crushing force.

Guided by sinusoidal corrugations, both straight and conical tubes symmetrically deformed in a progressive manner, and conical tubes folded sequentially from the top to the bottom of the tube (Figure 5). When *θ* ≥ 10°, the deformation mode of the corrugated conical tube transitioned from a stacking mode to a nesting mode (Figure 6). According to Wei et al. [38], the nesting mode has low energy absorption efficiency, and conventional conical tubes typically transition to the nesting mode only when *θ* ≥ 15°. This indicates that with the addition of corrugations, the nesting mode in conical tubes occurs at a smaller *θ* value, suggesting that the optimal *θ* range should be lower than that of conventional conical tubes. Corresponding to the progressive deformation pattern of each corrugated tube, its force–displacement curve (Figure 7) also fluctuated rhythmically. It can be seen in Figure 7 that the corrugated straight tube (CST) had the shortest compression displacement when densification occurred, and had the highest initial peak. The initial peak of the CST was also the highest within its effective deformation length, and the subsequent fluctuation amplitudes were small. The *IPCFs* of the CCTs were low by contrast, but their peaks gradually increased one by one. Compared with the *IPCF*, the increases in the crushing force value at the end peak when *θ* = 5°, 7.5° and 10° were approximately 20%, 45% and 53%, respectively. When *θ* = 12.5°, the end peak crushing force suddenly increased to twice as much as the *IPCF*. Therefore, the initial peak of the CCTs was often not the highest peak within the effective deformation length, which is consistent with the FEM results reported previously [30,31]. This should be an inherent feature of conically shaped tubes: the circular section of a conical tube is smaller in the upper part and larger in the lower part; thus, the top of the tube yields first with greater stress [39], and the resultant force in the lower part gradually increases as the section increases.

**Figure 8:**
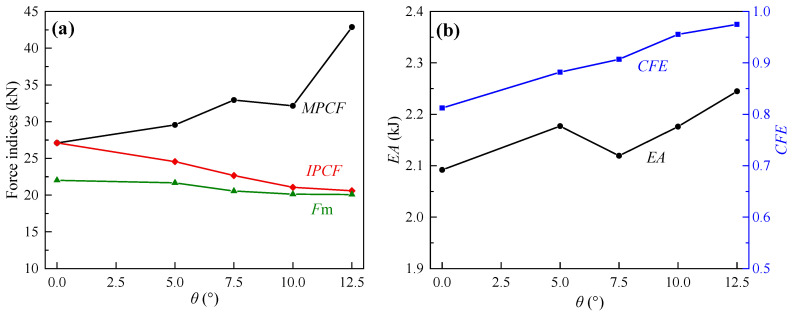
Comparison of crashworthiness indexes of CCTs. (**a**) *IPCF*, *MPCF* and *F*_m_; (**b**) *EA* and *CFE*.

From the perspective of crashworthiness indices (Figure 8), with the increase in *θ*, the *IPCF* and *F*_m_ of the CCTs showed a downward trend with SA0 as the highest point, but the decrease in *F*_m_ was small; the *MPCF*, on the other hand, increased rapidly with SA0 as the lowest point, especially from SA10 to SA12.5. The energy absorption ability and *CFE* of the CCTs were stronger than those of an equal mass CST. When *θ* = 5–10°, although the *EA* of SA7.5 was the smallest, the difference from SA5 and SA10 was not large. When *θ* > 10°, the *EA* of SA12.5 had a significant advantage over other CCTs due to a surge in the last peak crushing force. Meanwhile, the *CFE* of the CCTs continued to increase and gradually approached 1.0 with increasing *θ*.

In summary, both the CST and CCTs mentioned above deformed in an orderly manner according to predesigned corrugations. The *F*_m_ of the CCTs was close to that of the CST, but the *IPCF* was much smaller and the *CFE* and the *EA* were larger.

However, the problem is that their *MPCF* was higher than that of the CST, and there was a tendency to gradually increase peak amplitudes, especially in SA12.5, whose *MPCF* was significantly larger than the *IPCF* of itself and SA0. Although shifting the *MPCF* to the end of the crushing process can reduce the damage caused by the impact acceleration to the passengers and equipment in vehicles, which is beneficial to protecting life and property [40], it can still be dangerous if peak crushing forces continue to increase given that a crush happens quickly. The growth speed of the peak crushing forces of CCTs with *θ* = 5–10° was relatively slow, their *MPCF*s were relatively reasonable and were not much different from each other, and their *EA*s also changed little between each other. Therefore, it is comprehensively considered that CCTs with *θ* = 5–10° have better comprehensive crashworthiness; that is, by introducing corrugations, the optimal *θ* value of conical tubes can be reduced from 10–15° to 5–10°. To further consider the original intention of this paper, i.e., for the area occupied by the bottom of conical tubes, SA5 is the first choice to achieve the expected research goal. Taking SA5 as an example, compared to the corrugated straight tube, its *IPCF* decreased by 9.38%, while *CFE* and *EA* increased by 4.08% and 8.58%, respectively.

Nevertheless, if the *MPCF* of CCTs can be effectively reduced, their crashworthiness performance may be better and become a better choice. The above analysis has revealed that the increasing cross-sectional diameter of the conical structure is the main reason for the growth of peak crushing forces and the large *MPCF*. It is speculated that if the final part of the CCTs is shaped with a constant cross-sectional diameter to reduce the diameter of the bottom of the CCTs, the problem of a large *MPCF* may be solved. Thus, in the following, CCTs were modified based on this speculation (called modified CCTs), and the crashworthiness characteristics after modification were studied.

### 3.2. Crashworthiness Performance and Influence Mechanism of Modified CCTs

**Figure 9:**
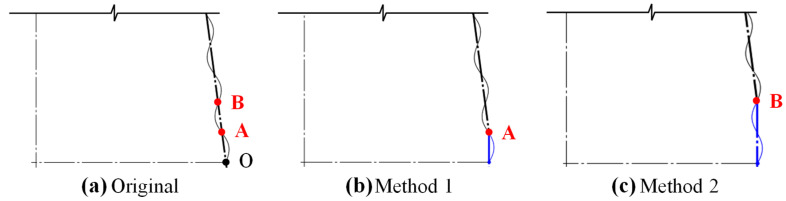
Illustration of bottom modification method (taking 7.5° CCT as an example). (**a**) Before modification: original CCT (SA7.5); (**b**) Method 1 (replacing OA): SA7.5_1_; (**c**) Method 2 (replacing OB): SA7.5_2_.

In this section, a scheme is proposed to reduce the bottom diameter of the CCTs by replacing the last one or two inclined half-wavelength corrugations of the original 5–10° CCTs (referred to as CCTs with *θ* = 5–10°, the same below) with vertical corrugations. That is, the two methods replace OA and OB in Figure 9a with vertical corrugations, respectively (Figure 9b,c). Subscripts 1 and 2 were added to the code of the original models for marking. For example, SA7.5_1_ is the modified 7.5° CCT using Method 1. In the following, the force–displacement curve and crashworthiness indices were analyzed first, and then the internal influence mechanism was examined based on deformation modes.

**Figure 10:**
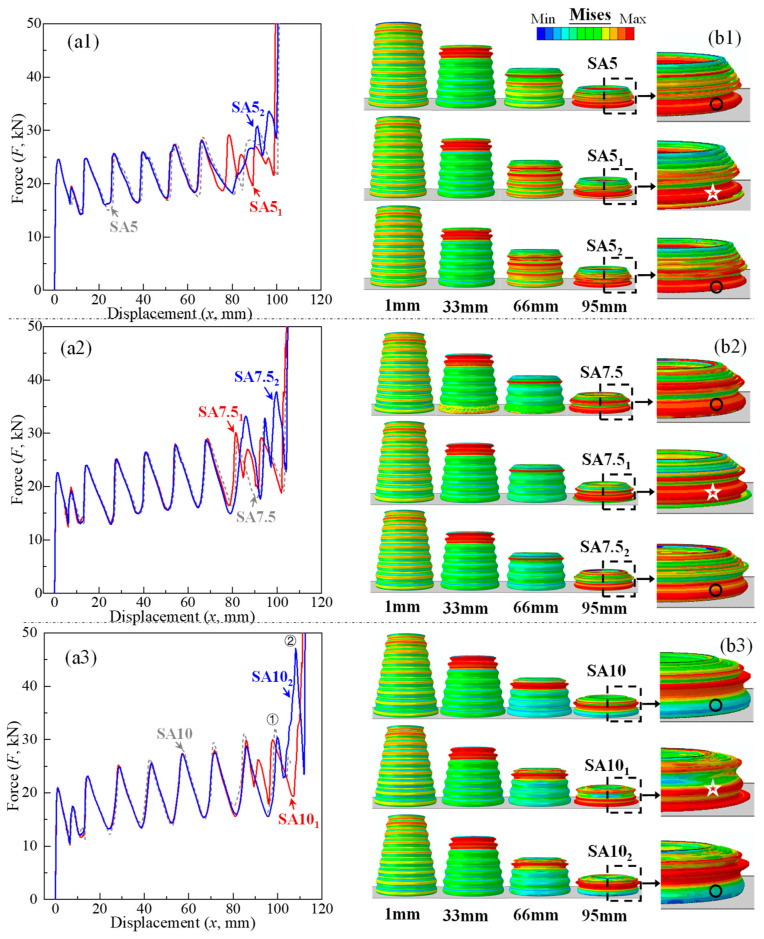
Force–displacement curves and deformation processes of modified CCTs. (**a1**,**b1**) 5°, (**a2**,**b2**) 7.5°, (**a3**,**b3**) 10°. Circles indicate the eighth corrugation folding last, while stars represent the seventh corrugation folding last, with the eighth corrugation deforming beforehand.

**Figure 11:**
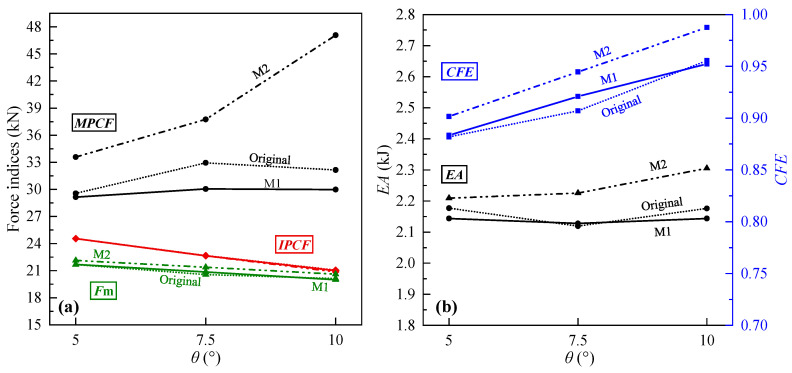
Comparison of crashworthiness indexes of modified CCTs. (**a**) *IPCF*, *MPCF* and *F*_m_; (**b**) *EA* and *CFE*. Note that M1 and M2 represent modification Methods 1 and 2, respectively.

Therefore, Figure 10a and Figure 11 show the force–displacement curves and crashworthiness indices of the modified CCTs, respectively. As seen in Figure 10a, the initial peaks of 5–10° CCTs before and after the modification almost overlapped (also Figure 11a, *IPCF*), but the plastic stage of the curves was different, especially after a displacement of 60 mm. For 5° and 7.5° CCTs (Figure 10(a1,a2)), Method 1 did not alleviate the trend of an increasing peak but lowered the end peak crushing force, resulting in a reduction in *MPCF* (Figure 11a, *MPCF*), while Method 2 led to an increase in *MPCF*. The effect of the two modification methods on the 10° CCT was more obvious, with the weakening of peak crushing forces starting from the fourth peak (Figure 10(a3)). The weakening effect of Method 1 lasted until the end, while Method 2 caused a surge in the peak crushing force at the end, and the increase was much higher than that of the 5° and 7.5° CCTs. A further analysis of Figure 11 shows that only Method 1 achieved the goal of reducing the *MPCF*, and it brought *F*_m_, *CFE* and *EA* close to those of the unmodified tubes. Method 2 was contrary to the original intention. Although the *EA* and *CFE* of each CCT were improved under Method 2, the improvement was mainly due to the increase in the end peak crushing force. Therefore, the final conclusion is that Method 1 had little effect on the improvement of the *MPCF* of SA5, which did not require modification. The effect on SA7.5 and SA10 was good, and their modified CCTs were better than the original CCTs.

**Figure 12:**
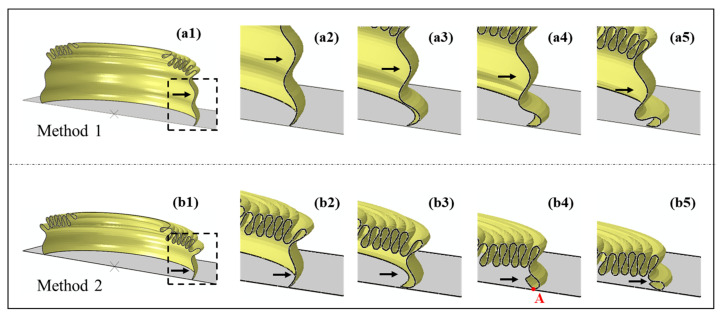
A detailed diagram of the deformation of the eighth corrugation (taking *θ* = 10° as an example). (**a1**–**a5**) SA10_1_; (**b1**–**b5**) SA10_2_. Among them, (**a1**,**b1**) are illustrations of the enlarged part, and the rest (**a2**–**a5**, **b2**–**b5**) display the deformation process of the enlarged part. (Note: the arrows in the figure refer to the last deformed corrugation.).

To reveal the reason why the two modification methods exhibited different effects, Figure 10b presents the deformation mode and stress contour of the modified CCTs. When the compression displacement did not exceed 66 mm, the corrugations of the modified CCTs still buckled one by one in order from top to bottom, and the stress distribution was also relatively consistent with that from before modification. When the compression displacement surpassed 66 mm, the deformation of the CCTs changed. The eighth corrugations of the CCTs modified with Method 1 folded before the seventh corrugations, becoming the penultimate buckled corrugation (Figure 10b, ☆). Although the sixth and seventh corrugations of the CCTs modified with Method 2 had different buckling sequences, their eighth corrugations were still the last to deform (Figure 10b, ○). Whether the eighth corrugation was the last to fold determined the effect of the modification method on reducing the *MPCF*. Taking SA10_1_ and SA10_2_ as examples (Figure 12), if the eighth corrugation was the last corrugated corrugation (SA10_2_, Figure 12b), then when the last corrugation buckled (corresponding to Figure 10(b3), ①), the tube took the contact point of the deformed last corrugation and the bottom plate as the fulcrum (Figure 12(b4), point A), forming a structure similar to diagonal bracing. Then, the tube continued to compress the deformation downward (Figure 12(b5)), generating a crushing peak ② higher than ① in Figure 10(a3) and leading to an increase in *MPCF*. However, if only peaks generated by the buckling of corrugations are considered (i.e., crushing peak ① is considered, but crushing peak ② is excluded), Method 2 also exhibited a good weakening effect on the end crushing peak of SA10, thus confirming that the sharp rise in the end peak crushing force of the original CCTs is indeed related to the bottom diameter and that reducing the bottom diameter is an effective way to solve the problem.

In summary, based on the idea of adjusting the bottom diameter, the countermeasure of “replacing the last one or two inclined half-wavelength corrugations with vertical corrugations” not only effectively approached the disadvantage of a large peak crushing force at the end of the crushing process but also further reduced the space occupied by the bottom of the conical tube and solved the current problem of conical tubes with good crashworthiness, laying the foundation for the development of new biomimetic single-cell and multi-cell plate–cone energy-absorbing boxes (tubes). Additionally, this study provides advice on the engineering application scenarios of the proposed modification methods for single-cell tubes (CCTs): (1) The 5° CCT occupied the smallest area at the bottom, and crashworthiness indices such as the *IPCF*, *EA* and CFE were obviously superior to those of the corrugated straight tube. Therefore, it already exhibited good practicability and crashworthiness and does not need modification. (2) For 7.5° and 10° CCTs, it is suggested to adopt Method 1 to effectively reduce the *MPCF* while keeping the indices of *F*_m_, *CFE* and *EA* basically unchanged.

**Figure 13:**
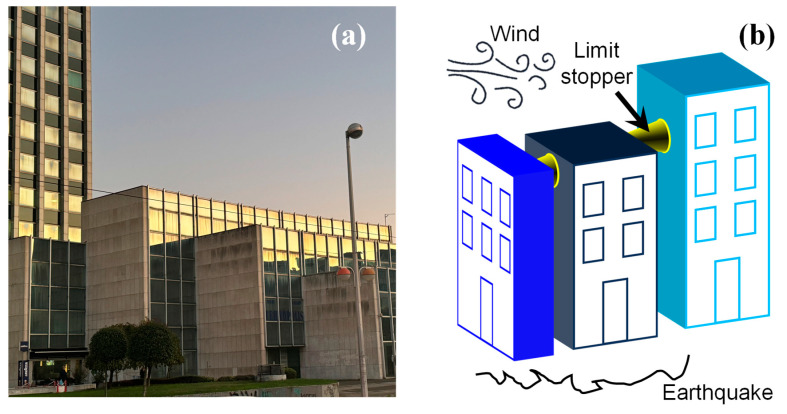
Application scenario of this study’s energy-absorbing device in densely built environments. (**a**) Real view of a building complex in Zagreb, Croatia (photo by the authors); (**b**) application schematic.

Based on this study, a solid foundation can be laid for creating another branch of the beetle elytron-inspired energy-absorbing structural system, providing new insights into the design of automotive crash boxes. Furthermore, in the architectural field, this study can be applied to limiting devices between the main building and podiums within densely built complexes (Figure 13). During events involving primarily horizontal forces, such as windstorms or earthquakes, it can effectively function as a replaceable sacrificial structure, dissipating energy through compression or tension by utilizing its elastoplastic strain energy to counteract the external work to prevent direct collision damage between buildings [41].

## 4. Conclusions

In this study, the crashworthiness performance of corrugated conical tubes (CCTs) with semi-apical angles (*θ*) of 0–12.5° and their modified tubes were investigated through numerical simulation. The main results are as follows:

(1) By adding corrugations, the optimal semi-apical angle range of conical tubes was reduced from 10–15° to 5–10°. The *F*_m_ of the 5–10° CCTs was close to that of the corrugated straight tube (CST), but the *IPCF* was significantly smaller with larger *CFE* and *EA*. Taking SA5 as an example, compared to the corrugated straight tube its IPCF decreased by 9.38%, while *CFE* and *EA* increased by 4.08% and 8.58%, respectively. However, CCTs with different *θ* values all exhibited a gradual increase in the peak crushing force and a much higher *MPCF* than that of the CST;
(2) Considering that the increasing diameter of CCTs from top to bottom is the major reason for the gradual growth of the peak crushing force, modification methods of “replacing the last one or two inclined half-wavelength corrugations with vertical corrugations at the bottom of CCTs” were proposed. Modification Method 1 effectively solved the problem of the large end peak crushing force of CCTs with a *θ* ≥ 7.5°. The reason for the different modification effects of the two modification methods was revealed to be the deformation of the last corrugation (eighth corrugation);
(3) The applicable scenarios of modification were given: the 5° CCT does not need modification; 7.5° and 10° CCTs can consider adopting Method 1 to reduce the *MPCF* with *F*_m_, *EA*, *CFE* and other crashworthiness indices basically unchanged;
(4) This study comprehensively and systematically verified the crashworthiness characteristics of CCTs with a relatively small semi-apical angle, as well as the internal influence mechanism of the semi-apical angle on CCTs, which not only lays the foundation for the development of a new type of biomimetic single-cell and multi-cell crush box (tube) but also provides a numerical analysis basis and direct guidance for the application of the corrugated conical tube itself.

## Figures and Tables

**Table 1:** Material properties of aluminum alloy [35].

Density	Young’s Modulus	Poisson’s Ratio	Yield Strength	Tensile Strength	Elongation
2700 kg/m^3^	68.5 GPa	0.33	179.67 MPa	241.83 MPa	9.98%

## Data Availability

The original contributions presented in this study are included in the article. Further inquiries can be directed to the corresponding author(s).
